# Early Life Adversity Predicts Mortality Risk Through Substance Use in Adulthood

**DOI:** 10.1093/geroni/igaf122.2511

**Published:** 2025-12-31

**Authors:** Sarah Miller, Meredith Willard, Nicholas Turiano

**Affiliations:** West Virginia University, Morgantown, West Virginia, United States; West Virginia University, Morgantown, West Virginia, United States; West Virginia University, Morgantown, West Virginia, United States

## Abstract

The lifespan effects of adverse childhood experiences (ACEs) on elevated mortality risk during adulthood are well established (Felitti et al., 1998). However, the mechanisms driving this association are less well understood. The self-medication hypothesis suggests that those experiencing ACEs could cope by using alcohol, tobacco, and drugs during adolescence and emerging adulthood. However, less is known how these behavioral patterns persist into adulthood and old age. Thus, we used data from 6,253 adults (aged 25-75) participating in the Midlife in the United States (MIDUS) study to determine if adults with higher ACEs were more likely to use substances, and subsequently be more likely to die. Mortality data was obtained from the National Death Index between 1995-2022 (deceased=2,229; Msurvival time=24.08 years). Number of alcoholic drinks consumed in an average drinking occasion during heaviest drinking year (M = 3.51), any illicit drug use (12.24% yes), and number of cigarettes smoked per day during heaviest smoking year (M = 16.92) were used as mediators of the ACE-mortality association using parallel mediation in a Cox modeling analysis. Higher ACEs and both alcohol and tobacco use were associated with an increased hazard of dying (p’s < .05), with both indirect effects also significant. Those exposed to ACEs drank more alcohol and smoked more cigarettes in adulthood, which contributed to their increased risk of death. Results reveal that ACEs have a lasting impact on health well into adulthood and that these behavioral pathways should be closely monitored among populations exposed to high levels of ACEs.

